# A multi-scale flow model for production performance analysis in shale gas reservoirs with fractal geometry

**DOI:** 10.1038/s41598-018-29710-1

**Published:** 2018-07-30

**Authors:** Lei Wang, Zhenzhen Dong, Xiang Li, Zunyi Xia

**Affiliations:** 1 ERE & BIC-ESAT, College of Engineering, Peking University, Beijing, 100871 China; 2 Energy Innovation Software Co. Ltd., Beijing, 100094 China

## Abstract

Shale gas reservoirs can be divided into three regions, including hydraulic fracture regions, stimulating reservoir volume regions (SRV regions), and outer stimulating reservoir volume regions (OSRV regions). Due to the impact of hydraulic fracturing, induced fractures in SRV regions are often irregular. In addition, a precise description of secondary fractures in SRV regions is of critical importance for production analysis and prediction. In this work, the following work is achieved: (1) the complex fracture network in the SRV region is described with fractal theory; (2) a dual inter-porosity flow mechanism with sorption and diffusion behaviors is considered in both SRV and OSRV regions; and (3) both multi-rate and multi-pressure solutions are proposed for history matching based on fractal models and Duhamel convolution theory. Compared with previous numerical and analytic methods, the developed model can provide more accurate dynamic parameter estimates for production analysis in a computationally efficient manner. In this paper, type curves are also established to delineate flow characteristics of the system. It is found that the flow can be classified as six stages, including a bi-linear flow regime, a linear flow regime, a transition flow regime, an inter-porosity flow regime from the matrix to the fractures in the inner region, inter-porosity flow regime from matrix to fractures in the outer region, and a boundary dominant flow regime. The effects of the fracture and matrix properties, fractal parameters, inter-porosity flow coefficients, and sorption characteristics on type curves and production performance were studied in detail. Finally, production performance was analyzed for Marcellus and Fuling shale gas wells, in the U.S.A. and China, respectively.

## Introduction

The successful exploitation of shale gas heavily depends on the combination of horizontal drilling, completions, and fracturing technology^1,2^. It has been proven that multi-stage fracturing horizontal wells constitute a very effective way to exploit low-permeability shale gas reservoirs. Due to the complex fracture network created in stimulated reservoir volume regions, it is a great challenge to accurately analyze production performance and evaluate post-fracture performance in such complex fractured reservoirs^3^.

Since 1972, many analytical and semi-analytical methods on production and flow behaviors in conventional gas and shale gas reservoirs have been utilized to simulate pressure transient behavior for horizontal wells with hydraulic fractures. Soliman *et al*.^4^ analyzed fluid flow mechanisms for multi-fractured horizontal wells and presented an approximate model to investigate the influence of fracture number, fracture orientation, fracture direction, fracture conductivity, and horizontal well location on flow behaviors. Larsen and Hegre^5^ presented two kinds of models for fractured horizontal wells with multiple fractures with finite conductivity: circular fractures with radial flow and vertical fractures with linear flow. For circular fractures, a cylindrical coordinate system is used with the perpendicular axis of the fracture coinciding with the z axis and the center of the fracture located at z = 0. The fracture size is given by the width, the well radius r_w_ and fracture outer radius r_f_. For a vertical fracture, a Cartesian coordinate system is used with the x axis chosen as vertical axis, the y axis coinciding with the axis of the wellbore, and the center of the fracture located at z = 0. The fracture size is given by the width, the fracture half-length x_f_ away from the wellbore, and the fracture half-length y_f_ along the wellbore. Guo and Evans^6^ presented a randomly distributed vertical fractures model to predict production performance, and generated type curves to estimate reservoir properties and fracture characteristics. Bin *et al*.^7^ presented an improved analytical solution for fractured horizontal wells with different fracture intensities to investigate the effect of fracture properties on flow characteristics. However, due to constant rate assumption, the model cannot be applied to interpret production data. Wang *et al*.^8^ proposed a semi-analytical model in tight oil reservoirs with fractal geometries, which was used to describe complex pore sizes and random fractures in porous media. Since sorption characteristics were not incorporated into their model, the model cannot consider the effect of sorption on production. A technique was introduced by Rbeawi and Tiab^9^ to interpret the pressure transient behavior of fractured horizontal wells. The distribution of hydraulic fractures could be longitudinal or transverse, vertical or inclined, symmetrical or asymmetrical. Brown *et al*.^10^ and Stalgorova and Mattar^11^ proposed a classical tri-linear flow model to simulate production performance of fractured horizontal wells in unconventional reservoirs. However, complex geometries and sorption characteristics were not taken into account in these studies, so that they were not suitable for production performance analysis in shale gas reservoirs.

The above studies^4–11^ mainly focused on fluid flow in primary fractures, and ignored fluid flow inside secondary fractures and matrix. Due to the influence of induced fractures, uniform fracture models were not applicable to describe heterogeneous SRV zones. Some scholars attempted to utilize numerical models with a discrete fracture network to describe secondary fractures in SRV zones^12–15^. Although the production performance of a complex fracture network can be discerned through the use of numerical methods, numerical simulation is less attractive and pragmatic due to the large amount of requisite knowledge and time-consumption during the history matching process^3^. In contrast, the analytical model is an alternative for accurately forecasting flow behaviors and calculation efficiency.

Fractal geometry has shown potential in the analysis of flow and transport properties in porous media. Katz and Thompson^16^ were the pioneers to find experimental evidence that indicated that the pore spaces of a set of sandstone samples were fractals and self-similar over three to four orders of magnitude in length. Fractal theory can also describe complex fracture shape in shale gas and is easy to apply to obtain analytical solutions. Chang and Yortsos^17^ introduced fractal theory into petroleum engineering, and reported that the permeable fractures embedded within the matrix would be arranged in a fractal dimension. Cossio *et al*.^18^ presented a model with fractal geometry to develop a rapid and accurate semi-analytical solution for flow in a single vertical fracture that fully penetrates a homogeneous infinite-acting reservoir. Yao *et al*.^19^ proposed a fractal double-porosity model for the transient flow analysis of fluids. Kong *et al*.^20^ developed the basic formulae of seepage velocity, permeability, and porosity in both porous and fractured fractal media. Wang *et al*.^3^ presented semi-analytical modelling of flow behavior in fractured media with fractal geometry. Zhang *et al*.^21^ proposed an approach for estimating the size and equivalent permeability of fractal SRV zones for vertically fractured wells in tight reservoirs.

The proposed fluid flow models with fractal geometry^3,16–21^ are convenient for conducting pressure transient analysis for understanding flow in complex reservoirs^22–25^. Random fractures will be produced during the process of hydraulic fracturing. Fractal theory can exhaustively describe the randomness of pore sizes and gas reservoir heterogeneity^24^. However, most of the previous models are only involved in the aspects of pressure transient analysis in tight reservoirs, while shale multi-scale flow characteristics are not considered^4–12^. Second, gas PVT is assumed to be constant, so that the final results will lead to an assessment of errors. However, gas PVT changes with pressure in the development of shale gas reservoirs. Third, the characteristics of fractal geometry in SRV regions are not reflected in their models^4–12^.

**Figure 1:**
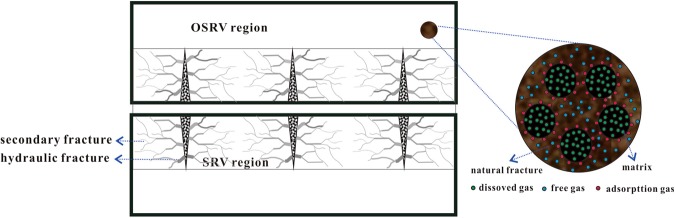
Schematic diagram of multiscale mechanism.

In this paper, an improved tri-linear flow model in shale gas reservoirs with fractal geometry is presented to analyze and predict production performance of multi-fractured wells. First, multi-scale flow mechanism in shale gas reservoirs was considered in the analytical model (Fig. 1). Second, the main fractures were considered into longitudinal rectangular fractures and secondary fractures in the SRV zone were described using fractal theory. Third, gas PVT changed with reservoir pressure in this paper, and a multi-rate solution with variable pressure was also proposed to interpret real well production data measured under various production systems. Finally, the downhill Simplex method was employed to form the history matching procedure.

## Methodology

### Conceptual model

Figure 1 presents the conceptual model of stimulated reservoir volume (SRV) with fractal geometry. Many authors have presented analytical models to investigate regular fracture networks, such as the single planar hydraulic fractures network and orthogonal hydraulic fractures network^4–15^.

**Figure 2:**
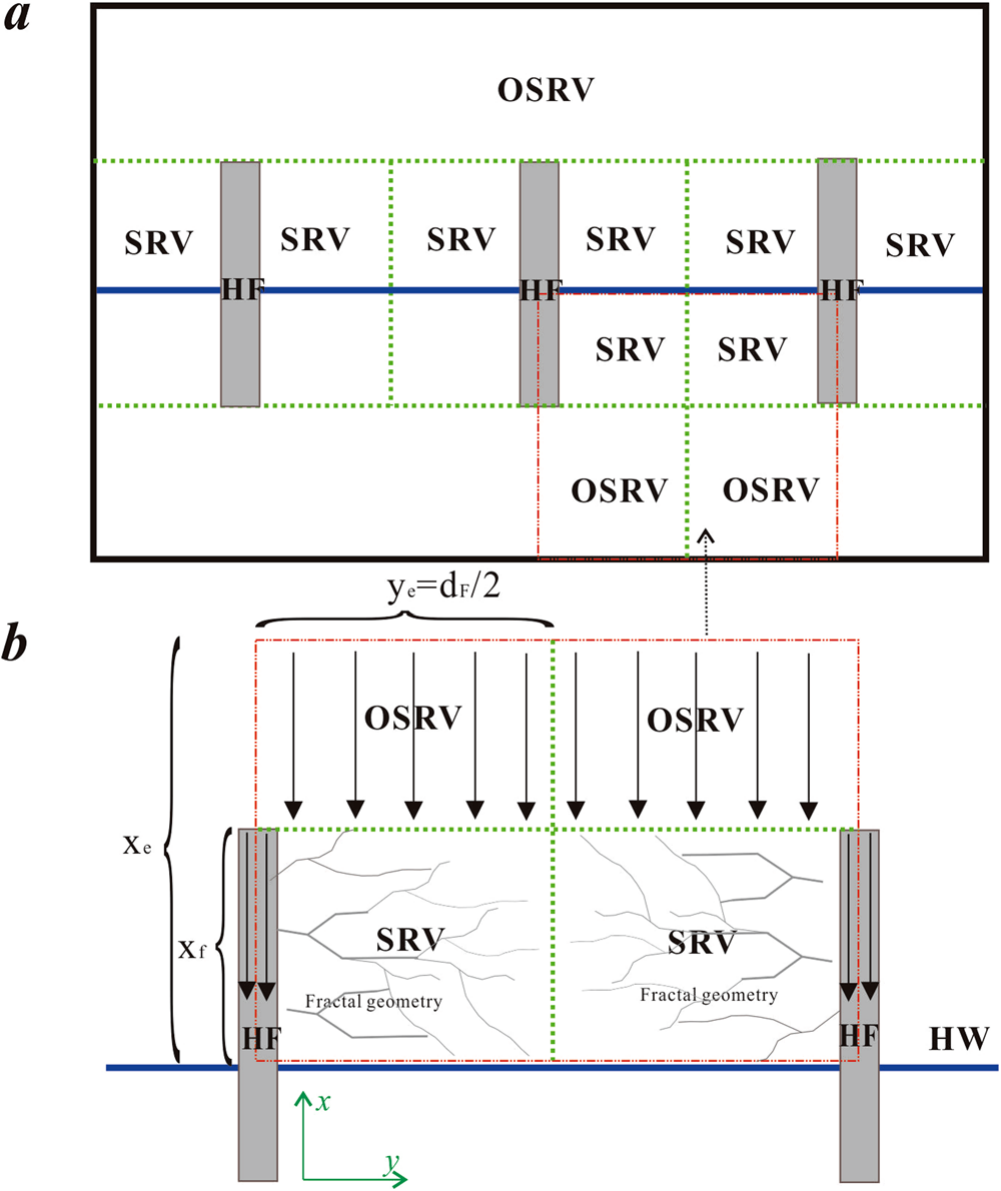
Schematic of flow: (**a**) flow regions in multi-stage fractured horizontal well; (**b**) flow unit with SRV region and OSRV region.

A schematic diagram of the presented model is shown in Fig. 2. Shale gas flow can be divided into three flow regions: flow in hydraulic fractures defined as region 1 (hydraulic fracture region); flow in the SRV region defined as region 2 (inner region); and flow in the OSRV region defined as region 3 (outer region). To establish mathematical models, the following basic assumptions are made:

1. Darcy flow occurs in hydraulic fractures;
2. Random fractures in SRV regions are considered using fractal geometry;
3. The OSRV region could be homogenous or double porosity with sorption behaviors;
4. Sorption characteristics are considered in the rock matrix;
5. The compressibility factor and viscosity change with pressure;
6. The Z-factor changes with pressure;
7. Knudsen diffusion and slippage effects are considered in the matrix model.

### Model establishment

Different from conventional reservoirs, shale gas seepage often exhibits strong nonlinearity^8^. In addition, physical parameters, such as the viscosity, compressibility factor and deviation factor, are functions of pressure. However, these methods are effective for only specific problems. The viscosity, compressibility and deviation factor of shale gas change with pressure, which leads to a non-linear seepage equation. The pseudo-time and pseudo-pressure^26–28^ are defined and a detailed deduction is provided in Appendix A of the Supplementary File. These definitions can be written in the following forms:

Pseudo-pressure:

1 $$m(p)=\frac{{\mu }_{i}{Z}_{i}}{{p}_{i}}{\int }_{0}^{p}\frac{p}{\mu Z}\,dp$$

Pseudo-time:

2 $${t}_{a}={\int }_{0}^{{\rm{t}}}\frac{{\mu }_{gi}{c}_{ti}}{{\mu }_{g}(p){c}_{t}(p)}dp$$

where *p* is pressure, Pa; *μ* is viscosity, Pa▪s; c_ti_ is initial total compressibility, Pa^−1^; *c*_t_ is total compressibility, Pa^−1^; t is time, s; *Z* is deviation factor; and *P*_i_ is initial pressure, Pa.

### Matrix model

Matrix inter-porosity flow occurs in both SVR regions and OSRV regions, as shown in Fig. 2. The spherical element was assumed to flow in the matrix, and a comprehensive continuity equation with sorption can be formulated as:

3 $$-\frac{1}{{r}_{im}^{2}}\frac{\partial }{\partial {r}_{im}}({r}_{im}^{2}{\rho }_{im}{v}_{im})=\frac{\partial ({\rho }_{im}{\varphi }_{im})}{\partial {t}_{a}}+{\rho }_{sc}\frac{\partial {V}_{i}}{\partial {t}_{a}}$$

where *V*_i_ is sorption volume, m^3^/m^3^; *ρ*_im_ is gas density in the matrix, kg/m^3^; *ρ*_sc_ is standard condition gas density, kg/m^3^; *v*_im_ is velocity in the matrix, m/s; *t*_a_ is pseudo-time, s. i = o, i can represent the outer reservoir (region 3) or inner reservoir (region 2), respectively. Pseudo-time *t*_*a*_ is defined in Eq. 2. The total compressibility in shale has been previously presented^29,30^ and can be expressed as:

4&x02010;1 $${c}_{t}=({c}_{g}[{S}_{gi}-{c}_{ep}]-\frac{\partial {c}_{ep}}{\partial p}+{c}_{d})$$

4&x02010;2 $${c}_{ep}=({c}_{f}+{c}_{w}{S}_{wi})({p}_{i}-p)$$

4&x02010;3 $${c}_{d}=\frac{{\rho }_{B}{B}_{g}{V}_{L}}{\varphi }(\frac{{p}_{L}}{{({p}_{L}+p)}^{2}})$$

where *c*_g_ is gas compressibility, Pa^−1^; *S*_gi_ is gas saturation; *c*_ep_ is intermediate variables; *c*_s_ is sorption compressibility, Pa^−1^; *c*_f_ is formation compressibility, Pa^−1^; *c*_w_ is water compressibility, Pa^−1^; *S*_wi_ is initial water saturation; *B*_g_ is gas volume coefficient; *V*_L_ is Langmuir volume, m^3^/kg; *p*_L_ is Langmuir pressure, Pa; *ρ*_B_ is density of shale, kg/m^3^; and *φ* is porosity. Accounting for gas slippage and gas diffusion, gas velocity in the matrix can be expressed as:

5 $${v}_{im}=-\,{k}_{im}{F}_{iD}\frac{\partial {p}_{im}}{\partial {r}_{im}}=-\,{k}_{im}[(1+8\alpha {k}_{n})+{\mu }_{g}{D}_{g}{c}_{g}/{k}_{im}]\frac{\partial {p}_{im}}{\partial {r}_{im}}$$

The equation of gas state is given as:

6 $${\rho }_{im}=\frac{{p}_{im}M}{nZRT}$$

Substituting Eq. 1, and Eqs 4 through 6 into Eq. 3 produces the governing equation of matrix:

7 $$\frac{1}{{r}_{im}^{2}}\frac{\partial }{\partial {r}_{im}}({r}_{im}^{2}{k}_{im}[(1+8\alpha kn)+\frac{{\mu }_{g}{D}_{g}{c}_{g}}{kim}]\frac{\partial {m}_{im}}{\partial {r}_{im}})={\varphi }_{im}{c}_{imt}\frac{\partial {m}_{im}}{\partial {t}_{a}}$$

and

8 $${c}_{imt}={c}_{g}+{c}_{im}+{c}_{id}$$

where *c*_im_ is matrix compressibility, Pa^−1^. The initial condition and boundary conditions can be given as:

9&x02010;1 $${{m}_{im}|}_{{t}_{a}=0}={m}_{i}({p}_{i})$$

and

9&x02010;2 $${{m}_{im}|}_{{r}_{im}=R}={m}_{if}$$

9&x02010;3 $${\frac{\partial {m}_{im}}{\partial {r}_{im}}|}_{{r}_{im}=0}=0$$

Equation 7 through 9 constitute the matrix seepage governing equation in both the SRV and OSRV regions. The dimensionless equations and solution method are provided in Appendix B.

### Natural fracture model of the OSRV region (region 3)

**Figure 3:**
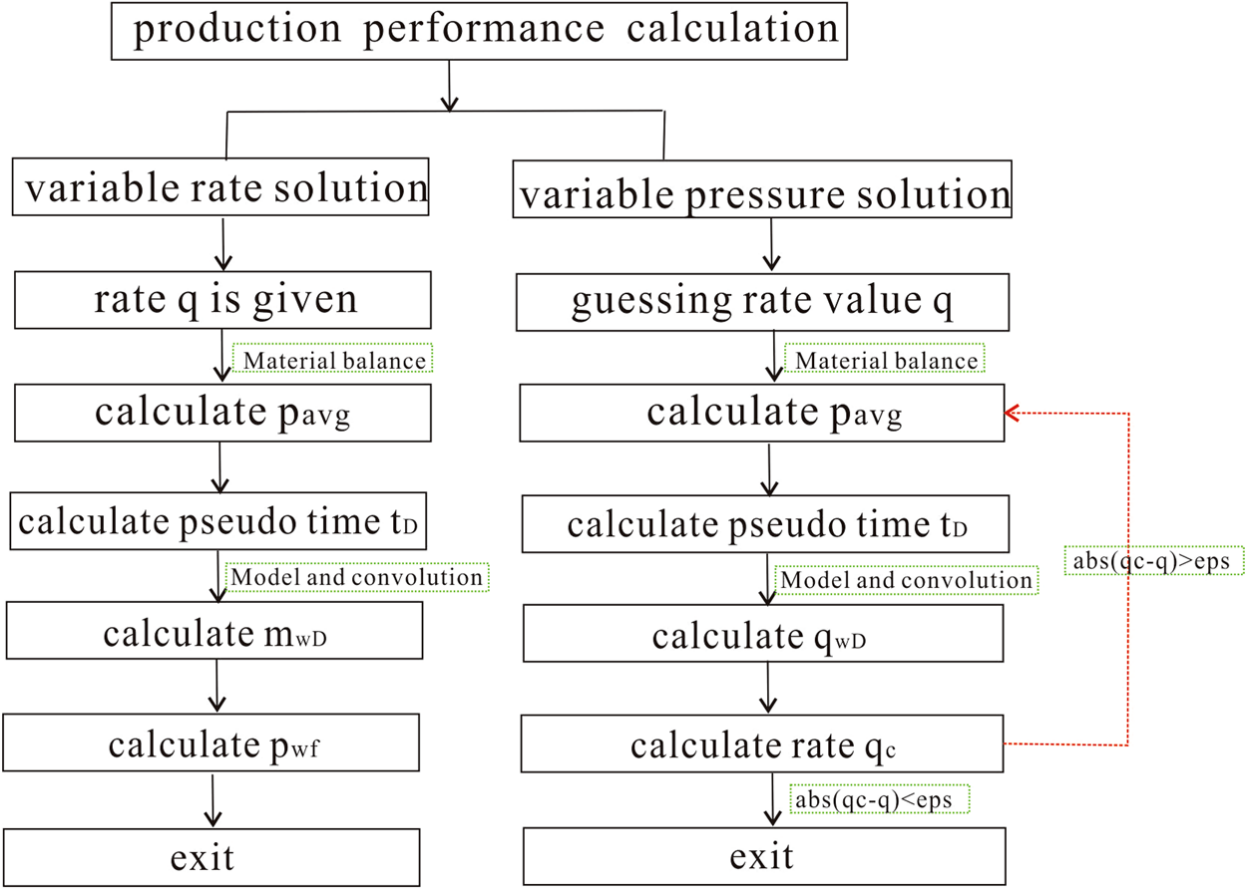
Calculation procedure for production performance analysis.

According to the above definitions of pseudo-pressure and pseudo-time, assuming one-dimensional flow in the *x* direction, as shown in Fig. 3, the diffusivity equation and the associated boundary conditions for the outer reservoir are given by:

10 $$\frac{{\partial }^{2}{m}_{of}(p)}{\partial {x}^{2}}=\frac{{\varphi }_{of}{c}_{oft}{\mu }_{gi}}{{k}_{of}}\frac{\partial {m}_{of}(p)}{\partial {t}_{a}}+\frac{3}{R}\frac{{k}_{om}{F}_{oD}}{{k}_{of}}{\frac{\partial {m}_{om}(p)}{\partial {r}_{om}}|}_{{r}_{om}=R}$$

11 $${(\frac{\partial {m}_{of}(p)}{\partial x})}_{x={x}_{e}}=0$$

12 $${m}_{of}{(p)}_{x={x}_{f}}={m}_{If}{(p)}_{x={x}_{f}}$$

where *k*_of_ is the fracture permeability in the outer region, m^2^; *φ*_of_ is fracture porosity in the outer region; *k*_om_ is matrix permeability in the outer region, m^2^; *x*_f_ is fracture half-length, m; *x*_e_ is boundary length, m; *c*_oft_ is total compressibility in the outer region, Pa^−1^; and *m*_of_ is fracture pseudo-pressure in the outer region, Pa. Eqs 10 through 12 constitute the natural fracture model of the OSRV region.

### Natural fracture model of the SRV region (region 2)

The change in porosity and permeability in the inner region are large and heterogeneous. Therefore, the fractal model can be utilized to describe SRV behavior. Furthermore, we work exclusively in Cartesian coordinates. Thus, the fractal relationships are given as^17,18^:

13&x02010;1 $${k}_{If}^{Fractal}(y)={k}_{If}{(\frac{y}{{x}_{f}})}^{d-\theta -2}={k}_{If}{y}_{D}^{d-\theta -2}$$

13&x02010;2 $${\varphi }_{If}^{Fractal}(y)={\varphi }_{If}{(\frac{y}{{x}_{f}})}^{d-2}={\varphi }_{If}{y}_{D}^{d-2}$$

Assuming one-dimensional flow in the *y* direction, the diffusivity equation is given by:

14 $$\begin{array}{rcl}\frac{\partial }{\partial y}({k}_{If}^{Fractal}\frac{\partial {m}_{If}(p)}{\partial y})+{(\frac{y}{{x}_{f}})}^{d-2}\frac{{k}_{If}}{{x}_{f}}({\frac{\partial {m}_{If}(p)}{\partial x}}_{x={x}_{f}}) & = & {(\frac{y}{{x}_{f}})}^{d-2}{\varphi }_{If}{c}_{Ift}\mu \frac{\partial {m}_{If}(p)}{\partial {t}_{a}}\\  &  & +\,{(\frac{y}{{x}_{f}})}^{d-2}\frac{3}{R}\frac{{k}_{Im}{F}_{ID}}{1}{\frac{\partial {m}_{\text{Im}}(p)}{\partial {r}_{Im}}|}_{{r}_{Im}=R}\end{array}$$

Substituting Eq. 13 into Eq. 14 yields:

15 $$\begin{array}{l}{(\frac{y}{{x}_{f}})}^{d-\theta -2}{k}_{If}[\frac{{\partial }^{2}{m}_{If}}{\partial {y}^{2}}+\frac{d-\theta -2}{y}\frac{\partial {m}_{If}}{\partial y}]+{(\frac{y}{{x}_{f}})}^{d-2}\frac{{k}_{If}}{{x}_{f}}({\frac{\partial {m}_{If}(p)}{\partial x}}_{x={x}_{f}})\\ \,={(\frac{y}{{x}_{f}})}^{d-2}{\varphi }_{If}{c}_{Ift}\mu \frac{\partial {m}_{If}(p)}{\partial t}+\,{(\frac{y}{{x}_{f}})}^{d-2}\frac{3}{R}\frac{{k}_{Im}{F}_{ID}}{1}{\frac{\partial {m}_{\text{Im}}(p)}{\partial {r}_{Im}}|}_{{r}_{Im}=R}\end{array}$$

The boundary conditions for the inner reservoir are given by:

16&x02010;1 $${(\frac{\partial {m}_{If}}{\partial y})}_{y={y}_{e}}=0$$

and

16&x02010;2 $${{m}_{If}|}_{y=\frac{\omega }{2}}={{m}_{F}|}_{y=\frac{\omega }{2}}$$

where *k*_If_ is the fracture permeability in the inner region, m^2^; *φ*_If_ is fracture porosity in the inner region; *k*_im_ is matrix permeability in the inner region, m^2^; *x*_f_ is fracture half-length, m; *c*_If_ is fracture compressibility in the inner region, Pa^−1^; *c*_Ift_ is total compressibility of the inner region, Pa^−1^; *m*_If_ is fracture pseudo-pressure of the inner region, Pa; *d* is fractal dimension representing the dimension of fractal fracture network embedded in the Euclidean matrix; and *θ* is connectivity index characterizing the diffusion process.

Eqs 15 and 16 are the natural fracture model of the OSRV region, and the solution method is presented in Appendix B.

### Hydraulic fracture model (region 1)

The flow in hydraulic fractures can be expressed as:

17 $$\frac{\partial ({k}_{F}\frac{\partial {m}_{F}(p)}{\partial x})}{\partial x}+{\frac{2{k}_{If}}{\omega }\frac{\partial {m}_{If}(p)}{\partial y}|}_{y=\frac{\omega }{2}}={\varphi }_{F}{c}_{F}{\mu }_{gi}\frac{\partial {m}_{F}(p)}{\partial {t}_{a}}$$

Inner boundary condition can be given as:

18&x02010;1 $${\frac{\partial {m}_{F}}{\partial x}|}_{x=0}=\frac{Q{\mu }_{gi}}{2wh{k}_{F}}$$

Outer boundary condition can be expressed as:

18&x02010;2 $${\frac{\partial {m}_{F}}{\partial x}|}_{x={x}_{f}}=0$$

where *Q* is flow rate, m^3^/s; *c*_F_ is fracture compressibility, Pa^−1^; *w* is fracture width, m; *φ*_F_ is hydraulic fracture porosity; and *k*_F_ is the fracture permeability, m^2^.

## Solution of Mathematical Model

### Bottom-hole pressure solution at constant rate conditions

A constant-rate solution can be obtained by using the Laplace transform method (Appendix B of the supplementary file), and we provide the solution of the non-linear difference equation using pseudo-pressure and pseudo-time definitions. The dimensionless definitions of the model and dimensionless solution at a constant rate condition are given as:

19 $${\bar{m}}_{FD}(p)=\frac{\pi }{{C}_{FD}s\sqrt{{\alpha }_{F}}}\frac{\cosh [\sqrt{{\alpha }_{F}}(1-{x}_{D})]}{\sinh (\sqrt{{\alpha }_{F}})}$$

where

20 $${\alpha }_{F}=2{\beta }_{F}/{C}_{FD}+s/{\eta }_{FD}$$

21 $${\beta }_{F}=-\,{(\frac{{w}_{D}}{2})}^{\gamma -1}\sqrt{{\alpha }_{{\rm{o}}}}\frac{{K}_{n-1}[\frac{\sqrt{{\alpha }_{o}}}{\gamma }{y}_{eD}^{\gamma }]{I}_{n-1}[\frac{\sqrt{{\alpha }_{o}}}{\gamma }{(\frac{{w}_{D}}{2})}^{\gamma }]-{I}_{n-1}[\frac{\sqrt{{\alpha }_{o}}}{\gamma }{y}_{eD}^{\gamma }]{K}_{n-1}[\frac{\sqrt{{\alpha }_{o}}}{\gamma }{(\frac{{w}_{D}}{2})}^{\gamma }]}{{I}_{n}[\frac{\sqrt{{\alpha }_{o}}}{\gamma }{(\frac{{w}_{D}}{2})}^{\gamma }]{K}_{n-1}[\frac{\sqrt{{\alpha }_{o}}}{\gamma }{y}_{eD}^{\gamma }]+{I}_{n-1}[\frac{\sqrt{{\alpha }_{o}}}{\gamma }{y}_{eD}^{\gamma }]{K}_{n}[\frac{\sqrt{{\alpha }_{o}}}{\gamma }{(\frac{{w}_{D}}{2})}^{\gamma }]}$$

22 $${\alpha }_{o}=\frac{{\beta }_{o}}{{C}_{RD}{y}_{eD}}+{u}_{I}$$

23 $${\beta }_{o}=\sqrt{{u}_{o}}\,\tanh [\sqrt{{u}_{o}}({x}_{eD}-1)]$$

24 $${u}_{I}=s[f(s)]=s[\frac{{\omega }_{I}}{{\eta }_{ID}}+\frac{{\lambda }_{I}{\eta }_{ID}}{5s}({\tau }_{I}\,\coth \,{\tau }_{I}-1)]$$

25 $${u}_{o}=s[g(s)]=s[\frac{{\omega }_{o}}{{\eta }_{oD}}+\frac{{\lambda }_{o}{\eta }_{oD}}{5s}({\tau }_{o}\,\coth \,{\tau }_{o}-1)]$$

In the above results, one-dimensional linear flow in the hydraulic fracture is considered. Flow choking within the fracture must be considered by using the following equation that was presented by Mukherjee and Economides^31^:

26 $${\bar{m}}_{FD}(p)=\frac{\pi }{{C}_{FD}s\sqrt{{\alpha }_{F}}}\frac{\cosh [\sqrt{{\alpha }_{F}}(1-{x}_{D})]}{\sinh (\sqrt{{\alpha }_{F}})}+\frac{{S}_{C}}{s}$$

S_C_ is flow skin, and expressed as:

27 $${S}_{C}=\frac{{k}_{If}h}{{k}_{F}{w}_{F}}[\mathrm{ln}(\frac{h}{2{r}_{w}})-\frac{\pi }{2}]$$

### Bottom-hole rate solution at constant pressure conditions

Note that the constant-pressure solution and constant-rate solution conform to the relation suggested by Van-Everdingen and Hurst^32^:

28 $${\bar{m}}_{FD}\times {\bar{q}}_{FD}=\frac{1}{{s}^{2}}$$

Therefore, the rate solution at a constant pressure condition can be obtained as:

29 $${\bar{q}}_{FD}=\frac{{C}_{FD}\sqrt{{\alpha }_{F}}}{s\pi }\frac{\sinh (\sqrt{{\alpha }_{F}})}{\cosh [\sqrt{{\alpha }_{F}}(1-{x}_{D})]}$$

### The solutions at variable rate or variable pressure conditions

During the process of production, both the rate and pressure change with the time. Constant rate and constant pressure solutions cannot be applied to a real reservoir. Through using convolution theory^29,33^, the variable rate and variable pressure solutions can be given by

30 $${{\rm{m}}}_{wD}({t}_{Dn})=\sum _{i=1}^{n-1}\frac{q({t}_{i})}{q({t}_{n})}[{{\rm{m}}}_{FD}({t}_{Dn}-{t}_{Di-1})-{{\rm{m}}}_{FD}({t}_{Dn}-{t}_{Di})]+{{\rm{m}}}_{FD}({t}_{Dn}-{t}_{Dn-1})$$

31 $${q}_{wD}({t}_{Dn})=\sum _{\begin{array}{c}i=1\\ n > 1\end{array}}^{n-1}{{\rm{m}}}_{FDi}[{q}_{FD}({t}_{Dn}-{t}_{Di-1})-{q}_{FD}({t}_{Dn}-{t}_{Di})]+{q}_{FD}({t}_{Dn}-{t}_{Dn-1})$$

### Calculation process

The material balance equation must be established to calculate pseudo-time and pseudo-pressure. The modified gas compressibility factor and material balance equation that were proposed by Moghadam^30^ for shale gas reservoirs were adopted in this paper.

The modified gas compressibility factor Z** is defined as:

32 $${Z}^{\ast \ast }=\frac{{\rm{p}}}{[\frac{1}{{S}_{gi}}\frac{p}{Z}({S}_{gi}-{c}_{ep}-{c}_{d})+\frac{{p}_{i}}{{Z}_{i}}(\frac{G}{{G}_{f}}-1)]\frac{{G}_{f}}{G}}$$

where the parameters *c*_*ep*_ and *c*_*d*_ are defined in Eq. 4. The modified material balance equation can be written as:

33 $$\frac{p}{{Z}^{\ast \ast }}=\frac{{p}_{i}}{{Z}_{i}^{\ast \ast }}(1-\frac{{G}_{p}}{G})$$

The average pressure can be determined by taking advantage of Eqs 32 and 33, and pseudo-pressure and pseudo-time can be calculated. The entire calculation process is illustrated in Fig. 3, which shows the procedures of variable rate solution and variable pressure solution. The procedures above could be applied to interpret production data from shale gas reservoirs.

### Validation of solution

**Figure 4:**
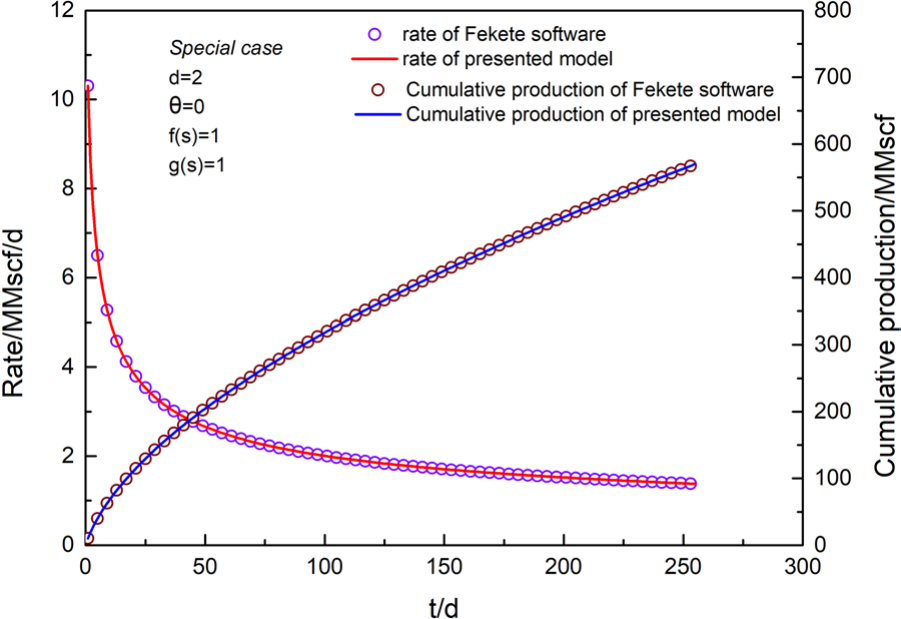
Validation of rate solution at constant pressure conditions for a special case (*d* = 2, *θ* = 0, *f*(*s*) = 1, *g*(*s*) = 1).

**Figure 5:**
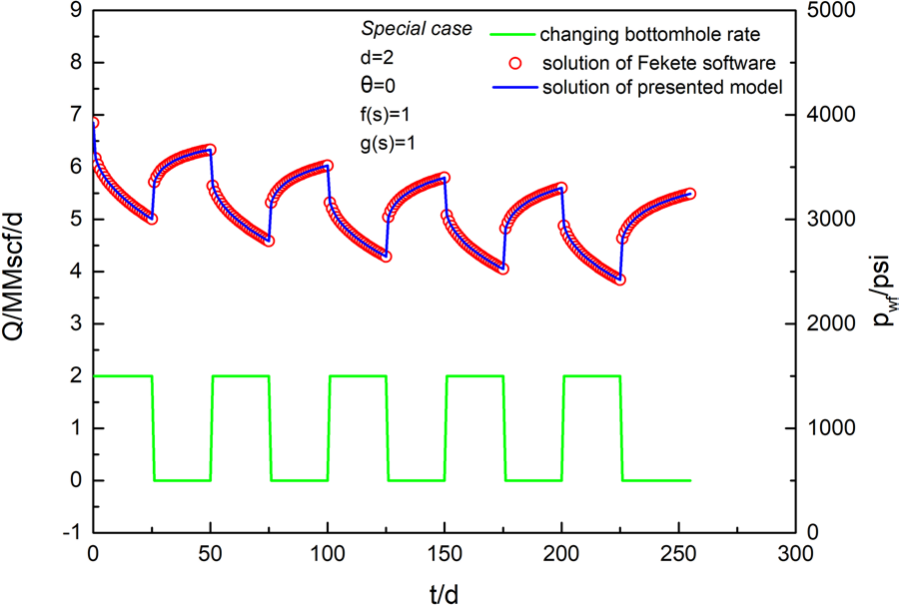
Validation of pressure solution at variable rate conditions for a special case (*d* = 2, *θ* = 0, *f*(*s*) = 1, *g*(*s*) = 1).

The accuracy of the coupled model was validated by comparing our results with those of an analytical solution using IHS Harmony software. The settings of the reservoir and fracture properties are listed in Table 1. We selected special cases of fractal parameters *d* = 2 and *θ* = 0, which represents constant permeability and constant porosity, as the fractal geometry has been not considered in commercial software. The total analysis time is 255 d. Figures 4 and 5 present the validation results by comparing the analytical results based on the proposed fractal model with the analytical solution from commercial software^10^. The algorithm from IHS Harmony software was presented by Brown *et al*.^10^ in 2009. A validation of the rate solution under a constant pressure condition of 2,000 psi is shown in Fig. 4. It is apparent that the production rate and cumulative gas production obtained from the proposed model agree very well with those from the analytical solution. A validation of the pressure solution under a variable rate condition is shown in Fig. 5. Figure 5 presents five cycles of open and shut well conditions. The pressure solution associated with a variable rate and the rate solution associated with variable pressure can be calculated using Eqs 30 and 31, respectively. The same parameters are listed in Table 1, and the production rate value for open well is set to 2 MMscf. Fractal parameters remain as *d* = 2 and *θ* = 0. The solution of the pressure under variable rate conditions is consistent with the solution from the commercial software IHS Harmony.

**Table 1:** Data for model validation.

Parameter name	Values	Units	Definitions
p_i_	3924	psia	Initial pressure
T	220	°F	Reservoir temperature
x_f_	171	ft	Fracture half-length
L	4175	ft	Horizontal well length
C_fD_	30	1	Fracture conductivity
n_f_	15	1	Fracture number
k_If_	1.0000E-03	md	Initial permeability of inner region
k_Of_	6.0000E-04	md	Permeability of outer region
h	150	ft	Formation thickness
φ_t_	7.1	%	Porosity
S_g_	66	%	Gas saturation
S_o_	0	%	Oil saturation
S_w_	34	%	Water saturation
C_f_	5.60E-06	1/psi	Pore compressibility factor
d	2	1	Fractal dimension
θ	0	1	Connectivity index
C_t_	2.01E-04	1/psi	Total compressibility factor
x_e_	4175	ft	Reservoir length
y_e_	700	ft	Reservoir width
r_w_	0.35	ft	Well radius
S_C_	0.0434	1	Choking skin
V_L_	200	scf/ton	Langmuir volume
P_L_	1200	psia	Langmuir pressure
ρ_B_	2.6	g/cm^3^	Shale rock density

## Results and Discussion

### Flow characteristics analysis of type curves

**Figure 6:**
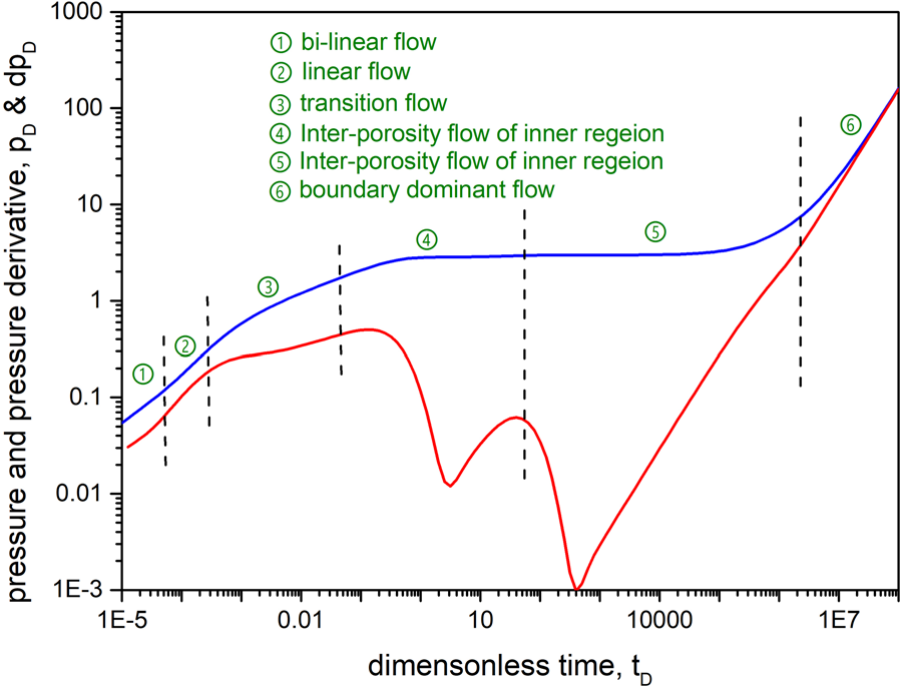
Flow characteristics of pressure and pressure derivative curves.

Flow characteristics analysis of pressure and pressure derivative curves is shown in Fig. 6 and the basic data used are shown in Table 2. It is apparent that the fluid flow in shale gas reservoirs can be classified into six stages:

1. The bi-linear flow regime is a straight line with a slope of 1/4 on the pressure derivative curve. The flow of gas occurs simultaneously both within hydraulic fractures and near them.
2. The linear flow regime is a straight line with a slope of 1/2 on the pressure derivative curve. In this period, linear flow around the fractures occupies the dominant position.
3. The transition flow regime (a) shows an indefinite slope line on the pressure derivative curve.
4. The first inter-porosity flow regime is a regime of supplementation from the shale matrix to the fracture system in the inner region. The derivative curve exhibits the first V-shaped segment.
5. The second inter-porosity flow regime represents the inter-porosity flow from the matrix system to the fracture system in the outer region. The pressure derivative curve shows the second V-shaped segment.
6. The boundary dominant flow regime is a straight line with a slope of 1 on the pressure derivative curve. In this regime, the process of inter-porosity flow is terminated, and the pressure between the matrix and fractures have increased to a state of dynamic balance.

**Table 2:** Dimensionless data for flow characteristics analysis.

Parameter name	Values	Units	Definitions
nf	1	Dimensionless	Fracture number
x_eD_	24	Dimensionless	Reservoir length
y_eD_	6	Dimensionless	Reservoir width
C_fD_	20	Dimensionless	Fracture conductivity
C_RD_	2	Dimensionless	Region conductivity
λ_I_	0.03	Dimensionless	Inter-porosity flow coefficient of inner region
λ_o_	3	Dimensionless	Inter-porosity flow coefficient of outer region
ω_I_	0.00001	Dimensionless	Permeability of outer region
ω_o_	0.000001	Dimensionless	Formation thickness
d	2.005	Dimensionless	Fractal dimension
θ	0	Dimensionless	Connectivity index

### Effect of sensitive factors on flow characteristics

**Figure 7:**
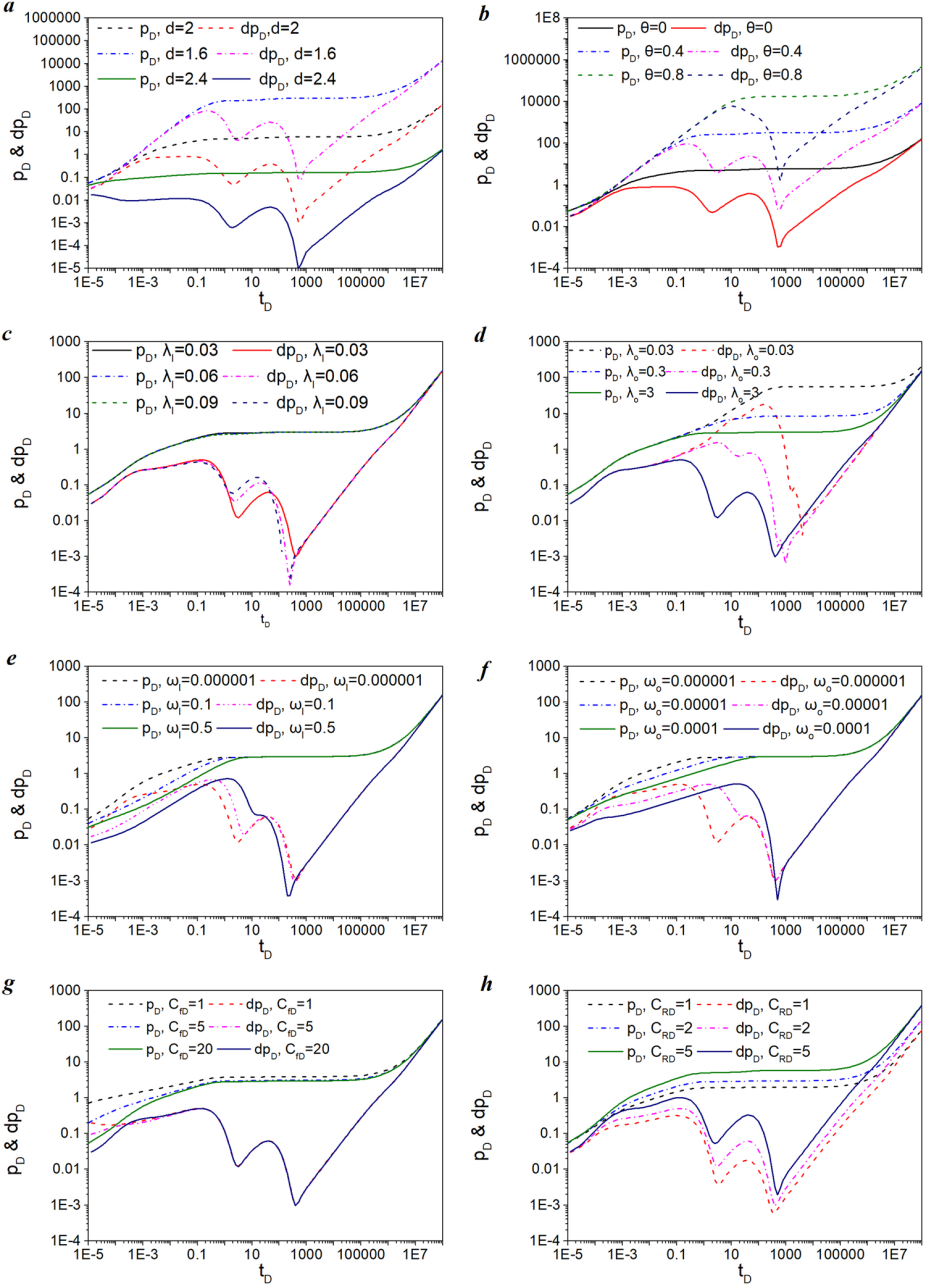
Influence of sensitive factors on flow characteristics: (**a**) fractal dimension; (**b**) connectivity index *θ*; (**c**) inter-porosity flow coefficient of inner region λ_I_; (**d**) inter-porosity flow coefficient of inner region λ_o_; (**e**) storage coefficient of inner region ω_I_; (**f**) storage coefficient of inner region ω_o_; (**g**) fracture conductivity C_fD_; (**h**) fracture half-length *x*_*f*_.

Figure 7 shows the influence of parameters, including the fractal dimension *d*; connectivity index *θ*; transfer coefficients of the inner region and outer region, *λ*_*I*_ and *λ*_*O*_, respectively; storage coefficients of the inner region and outer region, *ω*_*I*_ and *ω*_*O*_, respectively; and conductivity of fracture and region on pressure and pressure derivative curves. The same data are also listed in Table 2.

### Influence of fractal geometry parameters on type curves

Figure 7a presents the effect of fractal dimension *d* on plots of type curves, wherein fractal dimension d values are set to 1.6, 2, and 2.4. A larger *d* represents a more complex structure of fractal natural fractures. It is concluded that the fractal dimension *d* has a significant impact on flow characteristics within all periods. Dimensionless pressure *p*_*D*_ reflects pressure depletion during the production periods. From the pressure curve in Fig. 7a, it is indicated that a large fractal dimension will result in a small pressure depletion, which implies that the production rate will be enhanced under the same pressure drop. In addition, fracture morphology becomes more complex when fractal dimension *d* becomes large. Figure 7b presents the effect of the connectivity index of natural fractures in SRV region *θ* on plots of type curves. The *θ* values are set to 0, 0.4, and 0.8. Different from the fractal dimension, a large connectivity index will result in a large pressure depletion, which indicates that the production rate will be reduced under the same pressure drop. Moreover, a larger *θ* value leads to worse connectivity in the formation. In other words, pressure depletion is proportional to the connectivity index of natural fractures in the SRV region and is inversely proportional to fractal dimension.

### Influence of matrix transfer and storage parameters on type curves

Figure 7c through d show the effect of the inter-porosity coefficient between the matrix and natural fractures on type curves. It reflects gas flow ability from the matrix to natural fractures in the inner region. Similarly, the inter-porosity flow coefficient of outer region λ_o_ is proportional to matrix permeability k_om_ of the outer region, and is inversely proportional to fracture permeability k_of_ of the outer region. Thus, it reflects the gas flow ability from the matrix to natural fractures in the outer region.

**Figure 8:**
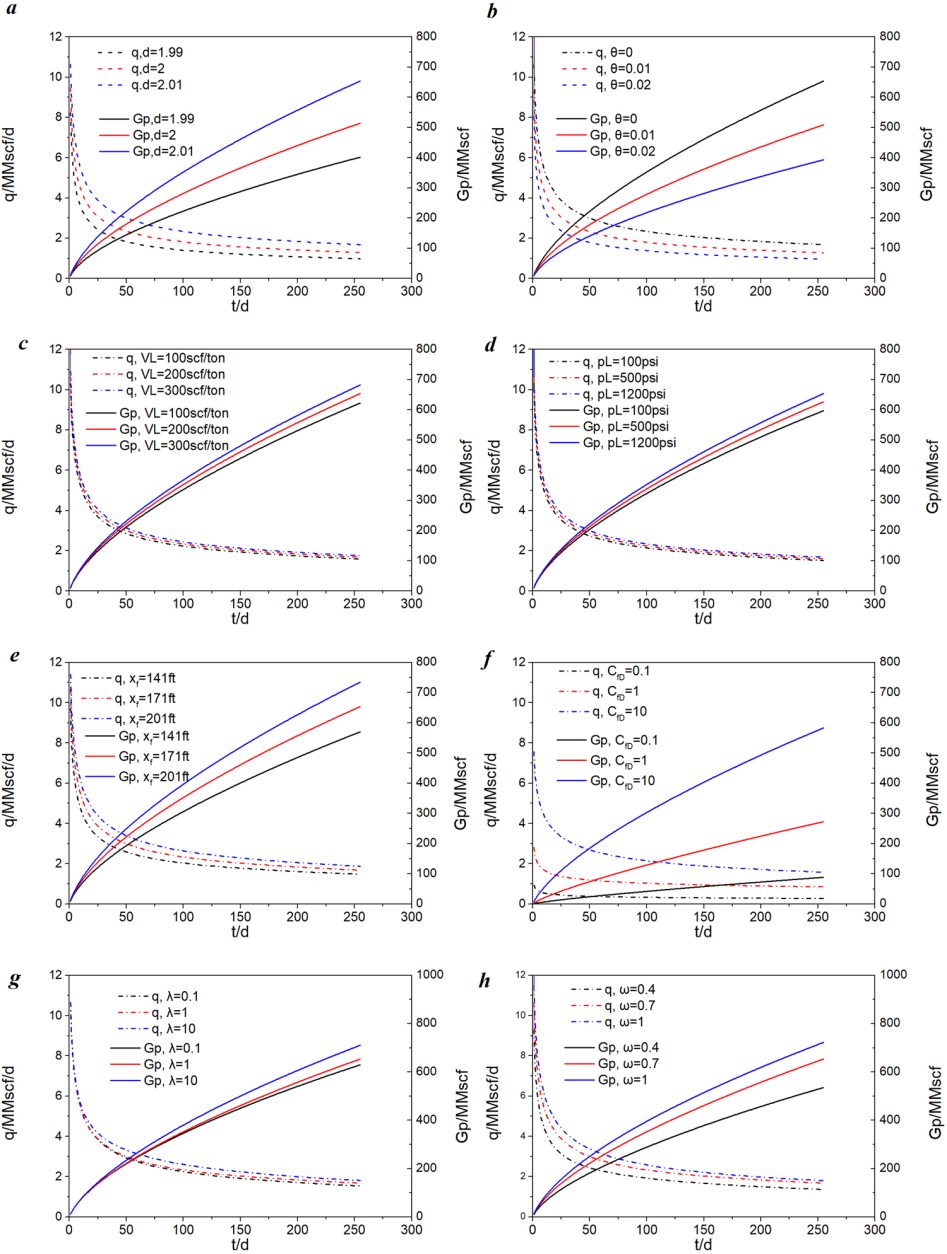
Influence of sensitive factors on production rate and cumulative production: (**a**) fractal dimension; (**b**) connectivity index *θ*; (**c**) Langmuir volume *V*_*L*_; (**d**) Langmuir pressure *p*_*L*_; (**e**) fracture half-length *x*_*f*_; (**f**) fracture conductivity *C*_*fD*_; (**g**) inter-porosity flow coefficient; (**h**) storage coefficient.

The mechanism of the theory reveals that a large inter-porosity flow coefficient reflects relatively strong matrix flow ability and weak fracture flow ability. Figure 7d presents the effect of the inter-porosity flow coefficient λ_o_ on type curves. The values are set to 0.03, 0.3, and 3. It is seen from Fig. 8d that a large λ_o_ value corresponding to strong matrix flow ability will lead to a small pressure depletion as the matrix compensates for pressure loss by supplying natural fractures. From the derivative curves of Fig. 7c and d, it is observed that flow in intermediate time is mainly affected by the inter-porosity flow coefficient.

Figure 7e and f show the effect of the storage coefficient of natural fractures on the type curves. The ω_I_ values reflect natural fracture energy, which was set to 0.000001, 0.1, and 0.5 in the inner region. The ω_I_ values reflect natural fracture energy and are set to 0.000001, 0.00001, and 0.0001in the inner region. It is known from the pressure curves of both figures that a large storage coefficient will lead to a small pressure depletion due to the storage of rich gas resources in the natural fractures. This results in an extension of the linear flow duration, which is indicated from the derivative curves of both figures. From the derivative curves of Fig. 7e and f, it could also be concluded that flow in early and medium time is mainly influenced by the storage coefficient.

### Influence of fracture conductivity and region conductivity on type curves

Figure 7g shows the effect of the hydraulic fracture conductivity on the type curves. The C_fD_ values reflect the flow ability of shale gas in hydraulic fractures and are set to 1, 5, and 20. It is observed from the pressure curves that a small fracture conductivity will lead to a large pressure depletion, which implies that the production rate will be enhanced by improving fracture conductivity. However, the production rate is improved only at an early time. It is apparent from the pressure derivative curves that there is no effect on flow in middle and late time.

Figure 7h presents the effect of the inner region conductivity *C*_*RD*_ on type curves. The *C*_*RD*_ values indicate flow ability of shale gas in the inner region and are set to 1, 2, and 5. *C*_*RD*_ has an important impact on fluid flow during all production periods. According to the definition of Eq. B-29, *C*_*RD*_ is proportional to the fracture permeability of the inner region and is inversely proportional to the fracture permeability of the outer region. The pressure curves in Fig. 7h show that a large region conductivity will lead to a large pressure depletion. It is apparent from the derivative curves that the shapes of the curves do not change as the inner region conductivity changes in the bi-linear flow period.

### Effect of sensitive factors on production performance

Figure 8 shows the effects of different parameters, including fractal dimension *d*, connectivity index *θ*, Langmuir volume *V*_*L*_, Langmuir pressure *P*_L_, fracture half-length *x*_*f*_, fracture conductivity *C*_*fD*_, inter-porosity flow coefficient of inner region and outer region *λ*_*I*_ and *λ*_*O*_, respectively, and storage coefficients of the inner region and outer region *ω*_*I*_ and *ω*_*O*_, respectively, on production rate and cumulative production. The data used are listed in Table 3. The bottom-hole pressure was set to 2,000 psi in all cases.

**Table 3:** Data for sensitive parameters analysis.

Parameter name	Values	Units	Definitions
p_i_	3924	psia	Initial pressure
T	220	°F	Reservoir temperature
x_f_	171	ft	Fracture half-length
L	4175	ft	Horizontal well length
C_fD_	30	1	Fracture conductivity
n_f_	15	1	Fracture number
k_If_	1.0000E-03	md	Initial permeability of inner region
k_Of_	6.0000E-04	md	Permeability of outer region
h	150	ft	Formation thickness
φ_t_	7.1	%	Porosity
S_g_	66	%	Gas saturation
S_o_	0	%	Oil saturation
S_w_	34	%	Water saturation
C_f_	5.60E-06	1/psi	Pore compressibility factor
d	2.01	1	Fractal dimension
θ	0	1	Connectivity index
λ_I_	1	1	Inter-porosity of inner region
λ_O_	1	1	Inter-porosity of outer region
ωI	0.7	1	Storage coefficient of inner region
ω_O_	0.7	1	Storage coefficient of outer region
C_t_	2.01E-04	1/psi	Total compressibility factor
x_e_	4175	ft	Reservoir length
y_e_	700	ft	Reservoir width
r_w_	0.35	ft	Well radius
S_C_	0.0434	1	Choking skin
V_L_	200	Scf /ton	Langmuir volume
P_L_	1200	psia	Langmuir pressure
ρ_B_	2.6	g/cm^3^	Shale rock density

### Fractal geometry

The fractal dimension values are set to 1.99, 2, and 2.01. Figure 8a presents the effect of fractal dimension *d* on plots of production rate and cumulative production versus time. A larger d represents a more complex structure of the fractal natural fractures. It is observed that the fractal dimension *d* has a significant impact on the solutions throughout the entire production period. A larger *d* value leads to a high production rate and a high cumulative production. In addition, as time increases, the rate first declines rapidly and then declines gradually.

Figure 8b presents the effect of the connectivity index of natural fractures in the SRV region *θ* on plots of production rate and cumulative production versus time. The *θ* values were set to 0, 0.01, and 0.2. The connectivity index reflects connectivity and tortuosity between natural fractures. It is apparent from Fig. 8b that the connectivity index of natural fractures in the SRV region has a significant impact on the solutions throughout the entire production period. As shown in Fig. 8b, a larger *θ* value leads to a low production rate and low cumulative production. This point implies that a larger *θ* reflects more complicated tortuosity, which will lead to worse connectivity in the formation.

### Sorption

Figure 8c presents the effect of the Langmuir volume on production behavior. The Langmuir volume *V*_*L*_ values are set to 100 scf/ton, 200 scf/ton, and 300 scf/ton. It is apparent from the figure that a larger *V*_*L*_ value will result in a high production rate and cumulative production. However, the increase in cumulative production is not obvious. The cumulative productions associated with *V*_*L*_ = 100 scf/ton, 200 scf/ton, and 300 scf/ton are G_p_ = 622.35 MMscf, 653.76 MMscf, and 682.26 MMscf, respectively. The fact that shale gas adsorption volume is directly proportional to the Langmuir volume will cause the shale gas adsorption volume to decrease as the Langmuir volume increases.

Figure 8d presents the effect of the Langmuir pressure *p*_*L*_ on plots of production rate and cumulative production versus time. The *p*_*L*_ values are set to 100 psi, 500 psi, and1200 psi. Simulation results show that production rate and cumulative production will increase as the Langmuir pressure increases. The fact that shale gas adsorption volume is inversely proportional to the Langmuir pressure will cause an increasing shale gas adsorption volume as the Langmuir pressure increases.

### Fracture parameters

Figure 8e shows the effect of fracture half-length on production behavior. The fracture half-length *x*_*f*_ was set to 141 ft, 171 ft, and 201 ft. It is apparent that a large *x*_*f*_ will lead to a high production rate and cumulative production. A larger *x*_f_ value has an important impact on enhancing production rate and cumulative production. The cumulative production of *x*_*f*_ = 141 ft, 171 ft, and 201 ft are G_p_ = 570.35 MMscf, 653.76 MMscf, and 734.77 MMscf.

Fracture conductivity also has an important impact on enhancing production rate and cumulative production. Figure 8f presents the effect of fracture conductivity on production behavior. The fracture conductivity *C*_*fD*_ values were set to 0.1, 1, and 10. It is apparent that a large *C*_*fD*_ value will lead to a high production rate and cumulative production. The cumulative production of *C*_*fD*_ = 0.1, 1, and 10 are G_p_ = 88.48 MMscf, 272.70 MMscf, and 583.31 MMscf, respectively. In the early stage, effectively increasing fracture conductivity effectively can remarkably improve the production rate and cumulative production.

### Matrix transfer and storage coefficients

Figure 8g presents the effect of the inter-porosity flow coefficient of the inner region on production behavior. The fracture inter-porosity flow coefficient *λ*_*I*_ values are set to 0.1, 1, and 10. It is apparent from the figure that a large *λ*_*I*_ value will lead to a high production rate and cumulative production. Figure 8h presents the effect of the storage coefficient of the inner region on production behavior. The fracture storage coefficient *ω*_*I*_ values are set to 0.4, 0.7, and 1. It is apparent from the figure that a large *ω*_*I*_ value will lead to a high production rate and cumulative production.

## History Matching of Production Data

### Automatic history matching

Automatic parameter estimation (APE) is a mathematical process, known as multi-variable optimization, which automatically adjusts a specified set of function parameters to minimize error between the function and measured data. Minimizing the mathematical function is called the objective function. With analytical modelling, the objective function is calculated as follows.

If the Calculate Pressure mode is used:

34 $${E}_{{\rm{avg}}}=\frac{{\sum }_{i}(\frac{|{{\boldsymbol{(}}{p}_{calc}{\boldsymbol{)}}}_{i}-{{\boldsymbol{(}}{p}_{hist}{\boldsymbol{)}}}_{i}|}{{{\boldsymbol{(}}{p}_{hist}{\boldsymbol{)}}}_{i}})}{{n}_{hist}}$$

If the Calculate Rate mode is used:

35 $${E}_{{\rm{avg}}}=\frac{{\sum }_{i}(\frac{|{{\boldsymbol{(}}{{\rm{q}}}_{calc}{\boldsymbol{)}}}_{i}-{{\boldsymbol{(}}{{\rm{q}}}_{hist}{\boldsymbol{)}}}_{i}|}{{{\boldsymbol{(}}{{\rm{q}}}_{hist}{\boldsymbol{)}}}_{i}})}{{n}_{hist}}$$

where *p*_*calc*_ is calculated pressure for the *i*-th point; *p*_*hist*_ is historical pressure for the *i*-th point; *q*_*calc*_ is calculated rate for the *i*-th point; *q*_*hist*_ is historical rate for the *i*-th point; *n*_*hist*_ is historical point number; and *E*_*avg*_ reflects the difference between the calculated and historical data. Therefore, a smaller *E*_*avg*_ indicates a better history match.

The Simplex routine^34^ is a non-linear regression algorithm used for APE for reservoir and well parameters (*k*_*If*_, *k*_*of*_, *x*_*f*_, *C*_*fD*_, *S*_*C*_, etc.) when modelling pressure and rate transient data. It requires only function evaluations of the objective function, and not the derivatives. Modification of the downhill Simplex method to achieve greater convergence is accomplished by imposing constraints on the parameters during the search. Estimates of the parameters are always checked against preset maximum and minimum values for each parameter. Once the routine has converged on some parameters, it is restarted with a slight perturbation away from the final values and is allowed to converge again. This ensures that the parameter estimates found are not the result of some local minimum in the residual, but rather a more global minimum.

### Field examples

#### Case 1: Marcellus shale, U.S.A

**Figure 9:**
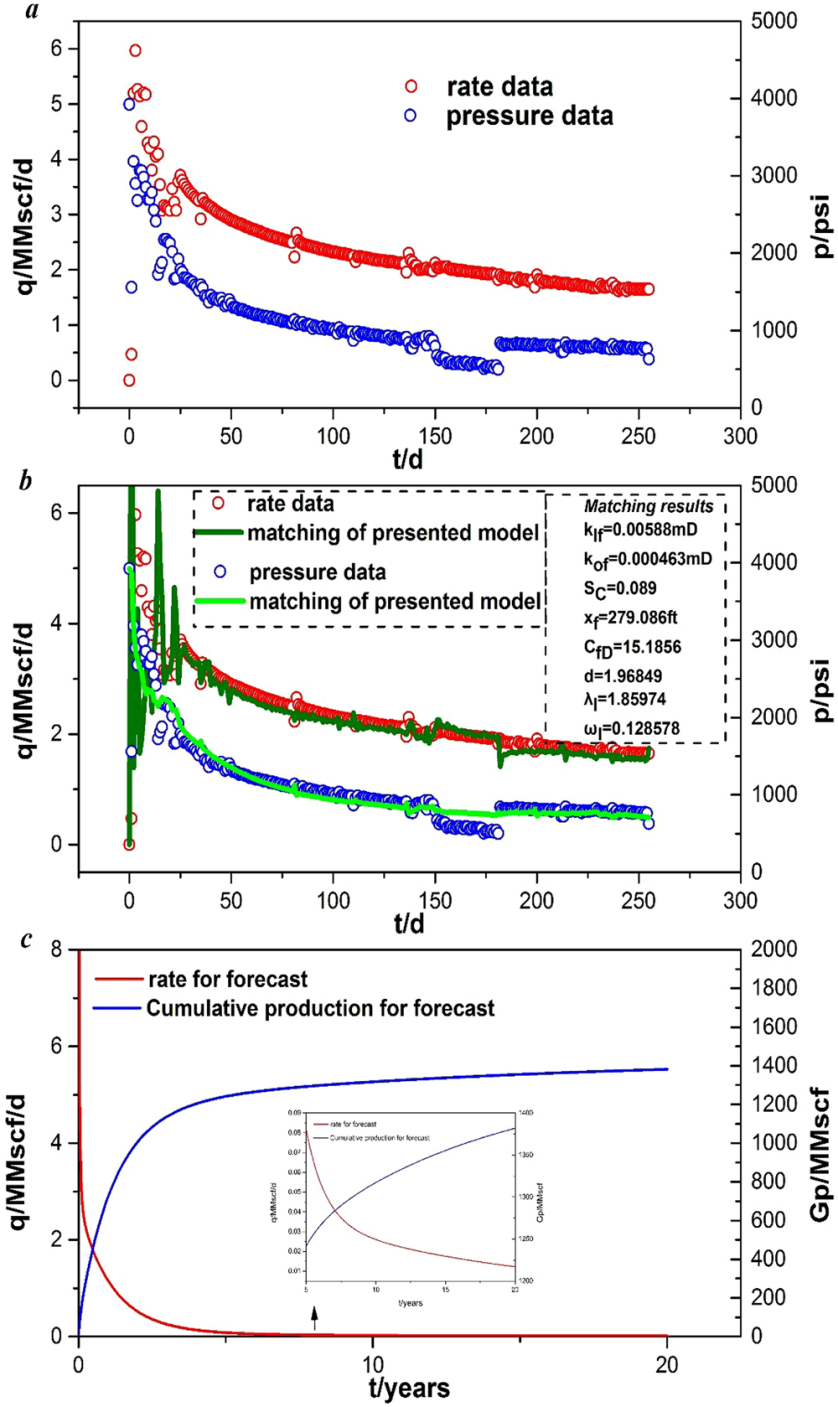
Field example from Marcellus shale gas wells in the U.S.A.: (**a**) real rate and pressure data; (**b**) history matching of production data; (**c**) forecast production performance.

Using our model, history matching of production data was performed based on field data. The well is a multi-stage fractured horizontal well with 12 fractures in a typical shale gas reservoir in the Marcellus shale gas field, U.S.A. The pressure data and rate data were acquired from production tests after almost one-year of production. Figure 9a shows the pressure data and rate data from Nov. 24^th^ 2009 to Aug 6^th^, 2010. The basic input parameters are summarized in Table 4. We selected the fracture permeability of the inner region, the fracture permeability of the outer region, choking skin, fracture half-length, fracture conductivity, fractal dimension, inter-porosity flow coefficient of inner region, and storage coefficient of inner region as uncertain parameters of history matching, which are listed in Table 4 and marked in bold.

**Table 4:** Basic data for Marcellus shale gas wells.

Parameter name	Values	Units	Definitions
p_i_	3924	psia	Initial pressure
T	220	°F	Reservoir temperature
**x**_**f**_	**120**	**ft**	**Fracture half-length**
L	4175	ft	Horizontal well length
**C**_**fD**_	**30**	**1**	**Fracture conductivity**
n_f_	12	1	Fracture number
**k**_**If**_	**1**.**0000E-03**	**md**	**Initial permeability of inner region**
**k**_**Of**_	**1**.**0000E-05**	**md**	**Permeability of outer region**
h	150	ft	Formation thickness
φ_t_	7.1	%	Porosity
S_g_	66	%	Gas saturation
S_o_	0	%	Oil saturation
S_w_	34	%	Water saturation
C_f_	5.60E-06	1/psi	Pore compressibility factor
**d**	**2**	**1**	**Fractal dimension**
θ	0	1	Connectivity index
**λ**_**I**_	**1**	**1**	**Inter-porosity of inner region**
λ_O_	1	1	Inter-porosity of outer region
**ωI**	**0**.**7**	**1**	**Storage coefficient of inner region**
ω_O_	0.7	1	Storage coefficient of outer region
C_t_	2.01E-04	1/psi	Total compressibility factor
x_e_	4175	ft	Reservoir length
y_e_	700	ft	Reservoir width
r_w_	0.35	ft	Well radius
**S**_**C**_	**0**	**1**	**Skin**
V_L_	200	scf/ton	Langmuir volume
P_L_	1200	psia	Langmuir pressure
ρB	2.6	g/cm3	Shale rock density

Figure 9b presents the historical matching results of production data from Marcellus shale gas wells using our proposed model. Our model fits the pressure and production rate very well. The presented model can also be applied to production performance analysis in complex conditions. Table 4 gives the initial estimation of uncertain parameters in shale gas, and the final matching results are shown in Fig. 9b. The *k*_*If*_ value is 0.00588 mD, the *k*_*Of*_ value is 0.000463 mD, the *S*_*C*_ value is 0.089, the *x*_f_ value is 279.086 ft, the *C*_*fD*_ value is 15.1856, the d value is 1.96849, the *λ*_*I*_ value is 1.85974, and the *ω*_*I*_ is 0.128578. The relative error of our model for all of the data is E_avg_ = 13.58%.

With the reservoir and fracture parameters determined by history matching, the production performance of the well can be predicted. In this case, the BHP was set to 631 psi from the final production data point. Figure 9c presents the predicted gas production and cumulative production. The cumulative production of the shale gas multi-fractured well with fractal geometry is estimated to be 1381.87 MMscf and the rate of well will become 0.01219 MMscf/d after 20 years. In the first five years, the rate of the well decreases rapidly, and cumulative production of the well increases dramatically. However, in the next 15 years, the rate of the well gradually decreased, and the cumulative production of the well increased slowly. From the fifth year to the twentieth year, the rate of well decreases only from 0.081876 MMscf/d to 0.01219 MMscf/d and cumulative production of well increases only from 1241.7 MMscf to 1381.87 MMscf, which does not constitute a remarkable improvement. In other words, it is not economically feasible to continue developing this shale gas well for up to 20 years.

#### Case 2: Fuling shale, China

**Figure 10:**
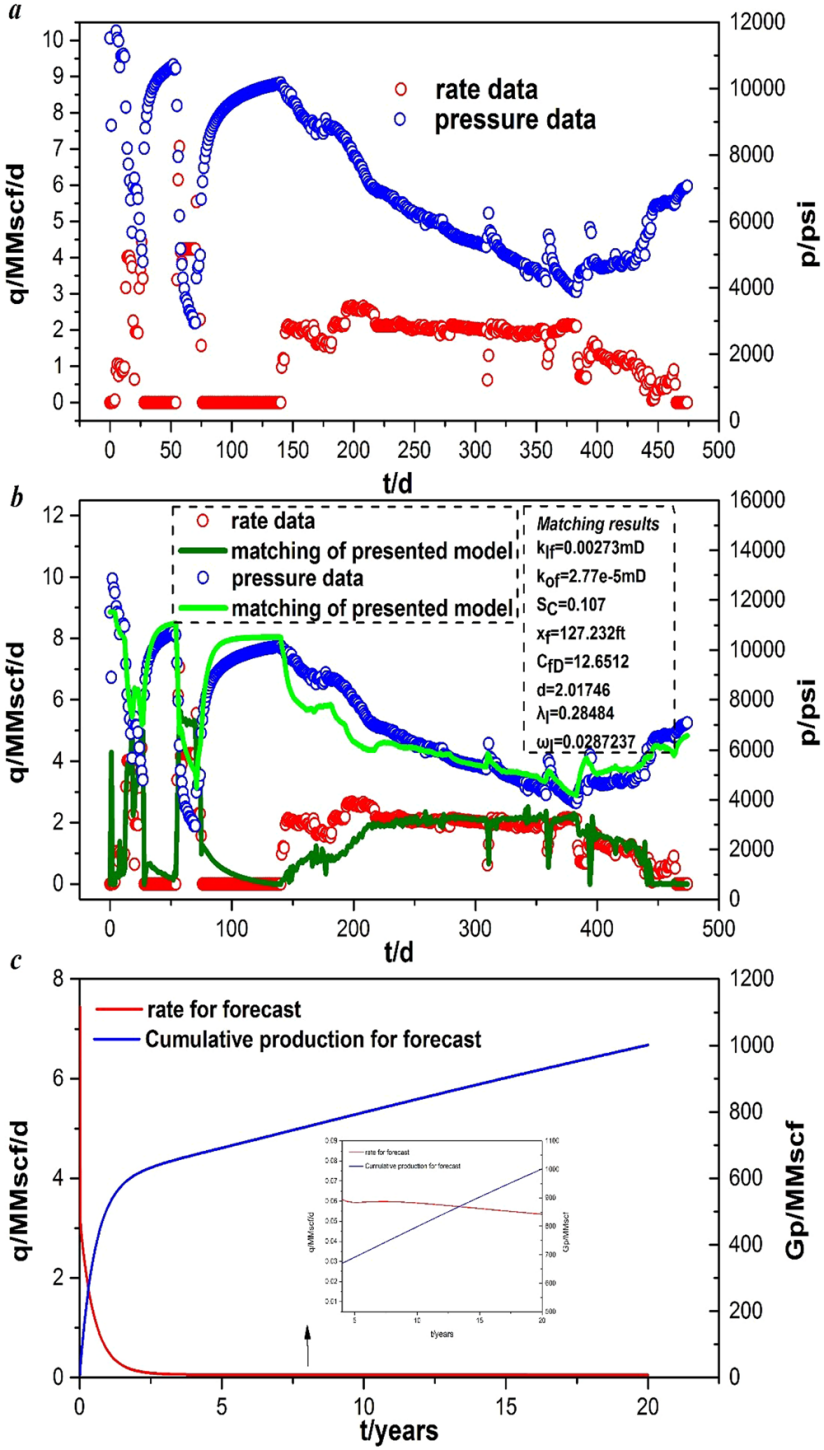
Field example from Fuling shale gas wells in China: (**a**) real rate and pressure data; (**b**) history matching of production data; (**c**) forecast production performance.

The second well is also a multi-stage fractured horizontal well with 21 fractures in a typical shale gas reservoir in the Fuling high pressure shale gas field, China. The pressure and production rate data were acquired from production test performed over 474 d. Figure 10a presents the pressure and production rate profile from Jan. 7^th^ 2016 to Apr. 24^th^, 2017. The basic input parameters are summarized in Table 5. The uncertain parameters of history matching are shown in Table 5 and are marked in bold.

**Table 5:** Basic data for Fuling shale gas wells.

Parameter name	Values	Units	Definitions
p_i_	11521.7	psia	Initial pressure
T	219.2	°F	Reservoir temperature
**x**_**f**_	**120**	**ft**	**Fracture half-length**
L	4926.56	ft	Horizontal well length
**C**_**fD**_	**30**	**1**	**Fracture conductivity**
n_f_	21	1	Fracture number
**k**_**If**_	**1**.**0000E-03**	**md**	**Initial permeability of inner region**
**k**_**Of**_	**1**.**0000E-05**	**md**	**Permeability of outer region**
h	136.12	ft	Formation thickness
φ_t_	3.71	%	Porosity
S_g_	80	%	Gas saturation
S_o_	0	%	Oil saturation
S_w_	20	%	Water saturation
C_f_	3.45E-07	1/psi	Pore compressibility factor
**d**	**2**	**1**	**Fractal dimension**
θ	0	1	Connectivity index
**λ**_**I**_	**1**	**1**	**Inter-porosity of inner region**
λ_O_	1	1	Inter-porosity of outer region
**ωI**	**0**.**7**	**1**	**Storage coefficient of inner region**
ω_O_	0.7	1	Storage coefficient of outer region
C_t_	5.01E-04	1/psi	Total compressibility factor
x_e_	4926.56	ft	Reservoir length
y_e_	1968	ft	Reservoir width
r_w_	0.164	ft	Well radius
**S**_**C**_	**0**	**1**	**Skin**
V_L_	80.7754	scf/ton	Langmuir volume
P_L_	869.565	psia	Langmuir pressure
ρB	2.6	g/cm3	Shale rock density

As shown in Fig. 10b, our model matched the BHP pressure and production rate very well from the Fuling shale gas field. Table 5 provides an initial estimation of uncertain parameters in the Fuling shale gas field, and the final matching results are shown in Fig. 10b. The *k*_*If*_ value is 0.00273 mD, the *k*_*Of*_ value is 2.77e-5 mD, the *S*_*C*_ value is 0.107, the *x*_f_ value is 127.232 ft, the *C*_*fD*_ value is 12.6512, the d value is 2.01746, the *λ*_*I*_ value is 0.28484, and the *ω*_*I*_ is 0.0287237. The relative error of our model for all of the data is E_avg_ = 4.16%.

According to the results, the production performance from Fuling shale gas wells can be predicted. In this case, if the BHP is set at 6948.28 psi, Fig. 10c shows the predicted gas production and cumulative production. The cumulative production of the shale gas multi-fractured well with fractal geometry is estimated to be 1001.43 MMscf after 20 years. In the first four years, the rate of the well decreases rapidly. However, in the next 16 years, the rate of well becomes approximately stable.

## Conclusions

In this study, a multi-scale flow model in shale gas reservoirs with fractal geometry was presented to investigate pressure transient response and analyze production performance, through which we examined the effects of shale rock properties and fractal characteristics. From the above analysis, the following conclusions are made:

1. The flow characteristics of type curves can be divided into six regimes: bi-linear flow regime, linear flow regime, transition flow regime, inter-porosity flow regime from the matrix to fractures in the inner region, inter-porosity flow regime from matrix to fractures in the outer region, and boundary dominant flow regime, respectively.
2. A large fractal dimension represents a complex structure of fractal natural fractures and leads to a small pressure depletion. A large connectivity index, which reflects connectivity and tortuosity among natural fractures, results in a large pressure depletion.
3. A large inter-porosity flow coefficient of the outer region and inner region corresponding to strong matrix flow ability will lead to a small pressure depletion because the matrix compensates for pressure loss by supplying natural fractures. A large storage coefficient results in a small pressure depletion due to rich gas resources that are stored by natural fractures.
4. A small fracture conductivity and a large region conductivity leads to a large pressure depletion.
5. A large fractal dimension, small connectivity index, large Langmuir volume, large Langmuir pressure, large fracture conductivity, large inter-porosity flow coefficient, and large storage coefficient can enhance the production rate and cumulative production of shale gas wells.
6. Based on a downhill Simplex algorithm, multiple uncertain parameters can be interpreted well by using the presented multi-rate solutions and multi-pressure solutions. The presented fractal model is well validated by matching real field examples.

## Electronic supplementary material

Supplementary Information
