# Supplementary material for: A multi-scale flow model for production performance analysis in shale gas reservoirs with fractal geometry
Source: Sci Rep. 2018 Jul 30;8:11464. doi: 10.1038/s41598-018-29710-1 (PMC6065449; doi:10.1038/s41598-018-29710-1)
Supplement: Supplementary file 1 — Supplementary Information [file 41598_2018_29710_MOESM1_ESM.doc]

**SUPPLEMENTAL MATERIALS**

**A multi-scale flow model for production performance analysis in shale gas reservoirs with fractal geometry**

**Lei Wanga,[[1]](#footnote-2) *, Zhenzhen Dong,^b^ Xiang, Li^a,b,*^, Zunyi, Xia^a^**

**Author Affiliations:** ^a^ BIC-ESAT, College of Engineering, Peking University, Beijing, China, 100871**.**  ^b^Energy Innovation Software Co. Ltd., Beijing 100094, China

**Corresponding Authors:**

BIC-ESAT, College of Engineering, Peking University, Beijing, China, 100083**.**  Phone Number: +86 13021906144; Fax Number: +8610-62751812.

E-mail: wanglei1986sp@foxmail.com.

**Running Title:**

**The Appendix** of the main article file named as "A multi-scale flow model for production performance analysis in shale gas reservoirs with fractal geometry".

**Conflict of Interest:**

We declare we have no competing interests.

**Appendix A. Linearization method of nonlinear equation**

Consider a two-dimensional nonlinear governing equation as follows:

(A-1)

Define the next integral form of pseudo-pressure and pseudo-time:

$m(p)=\frac{\mu_{i}Z_{i}}{p_{i}}\int_{0}^{p} \frac{p}{\mu Z}dp$ (A-2)

and

$t_{a}=\int_{0}^{\text{t}} \frac{\mu_{gi}c_{ti}}{\mu_{g}(p)c_{t}(p)}dp$ (A-3)

where *p* is pressure, Pa; μg is gas viscosity which changes with pressure, Pa▪s; c_ti_ is initial total compressibility, Pa^-1^; *c*_t_ is total compressibility, Pa^-1^; t is time, s; *Z* is deviation factor; *P*_i_ is initial pressure, Pa; *x*, *y* is spatial coordinates, m. Substituting Eq. A-2 and A-3 into Eq. A-1 yields:

(A-4)

Eq. A-4 is a linearized model for two-dimensional nonlinear flow. Thus, the nonlinear equation can be linearized by the definitions of pseudo-pressure and pseudo-time.

**Appendix B. Analytical solution for shale gas reservoirs**

***Solution of matrix in outer region and inner region.*** To obtain the solution of the outer region, we need to define the dimensionless reference diffusivity factor from Eq. 7 to Eq. 9:

(B-1)

(B-2)

and

$c_{ift}=c_{gi}+c_{if}$ (B-3)

where *c*_g_ is gas compressibility; and *c*_if_ is fracture compressibility. The dimensionless radial distance is defined as:

$r_{imD}=\frac{r_{im}}{R}$ (B-4)

The dimensionless time is defined by:

$t_{D}=\frac{\eta_{r}t_{a}}{x_{f}^{2}}$ (B-5)

We define the following diffusivity ratio:

$\eta_{iD}=\frac{\eta_{i}}{\eta_{r}}$ (B-6)

The storage coefficient and the inter-porosity flow coefficient are expressed as:

(B-7)

$\lambda_{i}=15\frac{k_{im}}{k_{if}}\frac{x_{f}^{2}}{R^{2}}$ (B-8)

The dimensionless pseudo-pressure could be given as:

$m_{imD}=\frac{2\pi k_{r}h}{B_{g}\mu Q}\left(m_{im}(p_{i})-m_{im}(p_{im})\right)$ (B-9)

where i=o and i represent outer region and inner region, respectively. Substituting Eqs. B-1 through B-9 into Eqs.7 through 9 yields:

$\frac{1}{r_{imD}^{2}}\frac{\partial}{\partial r_{imD}}\left(r_{imD}^{2}\frac{\partial m_{imD}}{\partial r_{imD}}\right)=\frac{15(1-\omega_{i})}{\lambda_{i}F_{iD}\eta_{iD}}\frac{\partial m_{imD}}{\partial t_{D}}$ (B-10)

The initial condition is given as:

$\left.m_{imD}\right|_{t_{D}=0}=0$ (B-11)

The boundary conditions are:

$\left.m_{imD}\right|_{r_{imD}=1}=m_{ifD}$ (B-12)

$\left.\frac{\partial m_{imD}}{\partial r_{imD}}\right|_{r_{imD}=0}=0$ (B-13)

Define Laplace transform as:

$\bar{m}_{imD}=\int_{0}^{\infty} m_{imD}e^{-st_{D}}dt_{D}$ (B-14)

where the over-bar symbol indicates dimensionless pressure in the Laplace-transform domain; and *s* is the Laplace-transform parameter with respect to dimensionless time, *t*_D_. Imposing the Laplace transform to the above equations, we can obtain the final solution:

$\bar{m}_{imD}=\frac{\sinh\left(r_{imD}\tau_{i}\right)}{r_{imD}\sinh(\tau_{i})}\left.\bar{m}_{ifD}\right|_{r_{imD}=1}$ (B-15)

where

$\tau_{i}=\sqrt{\frac{15(1-\omega_{i})s}{\lambda_{i}\eta_{iD}F_{iD}}}$ (B-16)

The derivative of Eq. B-15 at *r*_imD_=1 can be obtained as:

$\left.\frac{\partial\bar{m}_{imD}}{\partial r_{imD}}\right|_{r_{imD}=1}=\left(\tau_{i}\coth\tau_{i}-1\right)\left.\bar{m}_{ifD}\right|_{r_{imD}=1}$ (B-17)

***Solution of outer region (OSRV region).*** Define dimensionless variables as:

$x_{D}=\frac{x}{x_{f}}$ (B-18)

$y_{D}=\frac{y}{x_{f}}$ (B-19)

Substituting the above dimensionless variables into Eqs. 10 and 11, we have the following dimensionless equation:

$\frac{\partial^{2}m_{ofD}}{\partial x_{D}^{2}}\text{=}\frac{w_{o}}{\eta_{oD}}\frac{\partial m_{ofD}}{\partial t_{D}}+\frac{\lambda_{o}F_{oD}}{5}\left.\frac{\partial m_{omD}}{\partial r_{omD}}\right|_{r_{omD}=1}$ (B-20)

$\left(\frac{\partial m_{ofD}(p)}{\partial x_{D}}\right)_{x=x_{eD}}=0$ (B-21)

$m_{ofD}(p)_{x_{D}=1}=m_{IfD}(p)_{x_{D}\text{=}1}$ (B-22)

By performing the Laplace transform to Eqs. B-20 through B-22 and substituting Eq. B-17 into Eq. B-20, we have the following form:

$\frac{\partial^{2}\overline{m}_{ofD}}{\partial x_{_{D}}^{2}}-\frac{s\omega_{o}}{\eta_{oD}}\overline{m}_{ofD}-\frac{\lambda_{o}\eta_{oD}}{5}\left(\tau_{o}\coth\tau_{o}-1\right)\overline{m}_{ofD}\text{=}0$ (B-23)

Eq. B-23 can be rearranged as:

$\frac{\partial^{2}\overline{m}_{ofD}(p)}{\partial x_{_{D}}^{2}}-u_{o}\overline{m}_{ofD}(p)\text{=}0$ (B-24)

where

$u_{o}=s\left[g(s)\right]=s\left[\frac{\omega_{o}}{\eta_{oD}}+\frac{\lambda_{o}\eta_{oD}}{5s}\left(\tau_{o}\coth\tau_{o}-1\right)\right]$ (B-25)

Boundary conditions can be given as:

$\left(\frac{\partial\overline{m}_{ofD}(p)}{\partial x_{D}}\right)_{x=x_{eD}}=0$ (B-26)

$\overline{m}_{ofD}(p)_{x_{D}=1}=\overline{m}_{IfD}(p)_{x_{D}\text{=}1}$ (B-27)

The solution of the boundary value problem in Eqs. B-24 through B-27 can be obtained in the Laplace transform domain as follows:

$\overline{m}_{ofD}(p)=\overline{m}_{IfD}(p)_{x_{D}\text{=}1}\frac{\cosh\left[\sqrt{u_{o}}\left(x_{eD}-x_{D}\right)\right]}{\cosh\left[\sqrt{u_{o}}\left(x_{eD}-1\right)\right]}$ (B-28)

In Eq. B-28, the outer reservoir solution is given in terms of the inner reservoir pressure at the interface of the inner and outer reservoirs, *x*_D_=1.

***Solution of inner region (SRV region).*** Let:

$C_{RD}\text{=}\frac{k_{If}x_{f}}{k_{of}y_{e}}$ (B-29)

$y_{eD}\text{=}\frac{y_{e}}{x_{f}}$ (B-30)

According to dimensionless definitions of Eqs. B-9, B-29 and B-30, the dimensionless version of Eq. 15 can be written as:

(B-31)

The initial condition can be expressed as:

$\left.m_{IfD}\right|_{t_{D}=0}=0$ (B-32)

By imposing the Laplace transform to Eq. B-31, we can obtain:

(B-33)

Substituting Eq. B-17 into Eq. B-33, we have:

(B-34)

where

$u_{I}=s\left[f(s)\right]=s\left[\frac{\omega_{I}}{\eta_{ID}}+\frac{\lambda_{I}\eta_{ID}}{5s}\left(\tau_{I}\coth\tau_{I}-1\right)\right]$ (B-35)

Substituting from Eq. B-28, we obtain:

$\left.\frac{\partial\overline{m}_{ofD}}{\partial x_{D}}\right|_{x_{D}=1}=\left.-\beta_{o}\overline{m}_{IfD}\right|_{x_{D}=1}$ (B-36)

where

$\beta_{o}=\sqrt{u_{o}}\tanh\left[\sqrt{u_{o}}\left(x_{eD}-1\right)\right]$ (B-37)

Substituting Eqs. 35 through 37 into Eq. 34 yields:

(B-38)

with

(B-39)

The general solution of Eq. B-38 is:

(B-40)

where

, and (B-41)

We also note the useful pressure derivative:

(B-42)

The boundary conditions for the inner reservoir are given by:

(B-43)

and

(B-44)

Thus, the solution of Eq. B-40, with the boundary conditions given by Eqs. B-43 and B-44, is:

(B-45)

The pressure derivative can be written as:

(B-46)

***Final solution of hydraulic fracture region (HF region).*** Define:

$C_{FD}\text{=}\frac{k_{F}\omega}{k_{If}x_{f}}$ (B-47)

(B-48)

According to dimensionless variables defined above, **Eq. 17** becomes

$\frac{\partial^{2}m_{FD}(p)}{\partial x_{D}^{2}}+\left.\frac{2}{C_{FD}}\frac{\partial m_{IfD}(p)}{\partial y_{D}}\right|_{y_{D}=\frac{\omega_{D}}{2}}\text{=}\frac{1}{\eta_{FD}}\frac{\partial m_{FD}(p)}{\partial t_{D}}$ (B-49)

From Eq. B-46 we can substitute:

$\left.\frac{\partial\overline{m}_{IfD}}{\partial y_{D}}\right|_{y_{D}=\omega_{D}/2}=\left.-\beta_{F}\overline{m}_{FD}\right|_{y_{D}=\omega_{D}/2}$ (B-50)

where

$\beta_{F}=-\left(\frac{w_{D}}{2}\right)^{\gamma\text{-}1}\sqrt{\alpha_{\text{o}}}\frac{K_{n-1}\left[\frac{\sqrt{\alpha_{o}}}{\gamma}y_{eD}^{\gamma}\right]I_{n\text{-}1}\left[\frac{\sqrt{\alpha_{o}}}{\gamma}\left(\frac{w_{D}}{2}\right)^{\gamma}\right]\text{-}I_{n-1}\left[\frac{\sqrt{\alpha_{o}}}{\gamma}y_{eD}^{\gamma}\right]K_{n\text{-}1}\left[\frac{\sqrt{\alpha_{o}}}{\gamma}\left(\frac{w_{D}}{2}\right)^{\gamma}\right]}{I_{n}\left[\frac{\sqrt{\alpha_{o}}}{\gamma}\left(\frac{w_{D}}{2}\right)^{\gamma}\right]K_{n-1}\left[\frac{\sqrt{\alpha_{o}}}{\gamma}y_{eD}^{\gamma}\right]+I_{n-1}\left[\frac{\sqrt{\alpha_{o}}}{\gamma}y_{eD}^{\gamma}\right]K_{n}\left[\frac{\sqrt{\alpha_{o}}}{\gamma}\left(\frac{w_{D}}{2}\right)^{\gamma}\right]}$ (B-51)

Imposing the Laplace transform to Eq. B-49 with the solution given by Eqs. B-50 and B-51, Eq. B-49 becomes:

$\frac{\partial^{2}\overline{m}_{FD}(p)}{\partial x_{D}^{2}}-\alpha_{F}\overline{m}_{FD}(p)=0$ (B-52)

where

$\alpha_{F}=\frac{2\beta_{F}}{C_{FD}}+\frac{s}{\eta_{FD}}$ (B-53)

The boundary conditions of the hydraulic fracture problem are given by:

$\left.\frac{\partial\overline{m}_{FD}}{\partial x_{D}}\right|_{x_{D}=1}=0$ (B-54)

$\left.\frac{\partial\overline{m}_{FD}}{\partial x_{D}}\right|_{x_{D}=0}=-\frac{\pi}{sC_{FD}}$ (B-55)

Then, the dimensionless pressure solution for the hydraulic fracture is obtained as:

$\overline{m}_{FD}(p)\text{=}\frac{\pi}{C_{FD}s\sqrt{\alpha_{F}}}\frac{\cosh\left[\sqrt{\alpha_{F}}\left(1-x_{D}\right)\right]}{\sinh\left(\sqrt{\alpha_{F}}\right)}$ (B-56)

Eq. B-56 is the final solution of wellbore pseudo-pressure in the Laplace domain.

1. ***Corresponding authors. Correspondence requests for materials should be addressed to X. Li (email: [lixiangcn@pku.edu.cn](mailto:lixiangcn@pku.edu.cn), lixiangcn@126.com)or L. Wang (email: [wanglei1986sp@foxmail.com](mailto:wanglei1986sp@foxmail.com)). [↑](#footnote-ref-2)
